# Supplementary material for: Formation of the Atlantic Meridional Overturning Circulation lower limb is critically dependent on Atlantic-Arctic mixing
Source: Nat Commun. 2024 Aug 26;15:7341. doi: 10.1038/s41467-024-51777-w (PMC11347556; doi:10.1038/s41467-024-51777-w)
Supplement: Supplementary file 1 — Supplementary Information [file 41467_2024_51777_MOESM1_ESM.pdf]

Supplement to 'Formation of the Atlantic Meridional Overturning Circulation lower limb is critically dependent on Atlantic-Arctic mixing'

Dipanjan Dey[1,2*], Robert Marsh[1], Sybren Drijfhout[1,3], Simon A. Josey[4], Bablu Sinha[4], Jeremy Grist[4], and Kristofer Döös[5]

[1]School of Ocean and Earth Science, University of Southampton, Southampton, UK
[2]School of Earth, Ocean and Climate Sciences, Indian Institute of Technology Bhubaneswar
[3]Royal Netherlands Meteorological Institute, De Bilt, Netherlands
[4]National Oceanography Centre, Southampton, UK
[5]Department of Meteorology, Stockholm University, Stockholm, Sweden
[*]d.dey@soton.ac.uk

Supplementary Figures 1 – 7

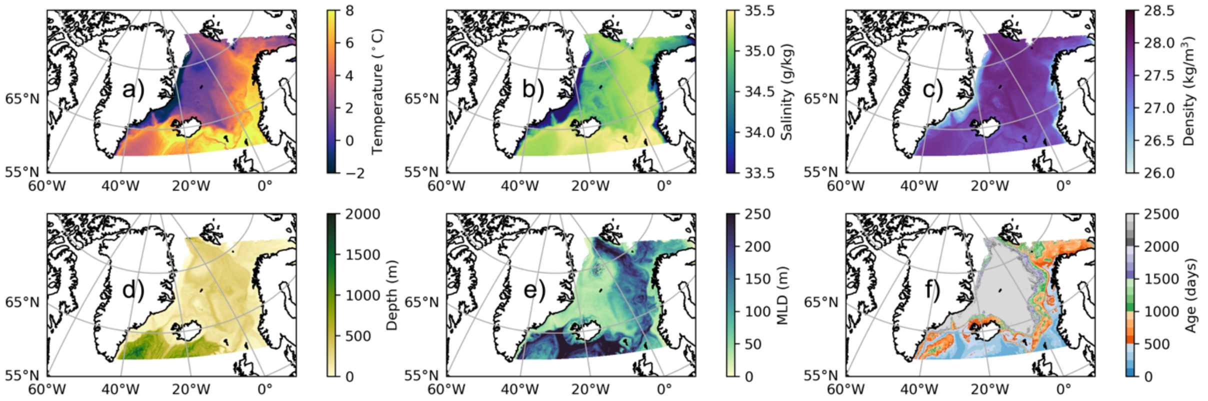

Supplementary Figure 1: a-f) Along-path water-mass properties obtained from Lagrangian trajectories that corresponds to Atlantic inflow. All trajectories were considered that have reached one of the three ending zones (as shown by red, blue and orange lines in Figure 1). All depth levels, associated with trajectories, are considered in the computation. The panels correspond to a) Conservative temperature (°C), b) absolute salinity (g kg^{-1}), c) potential density referenced to surface (kg m^{-3}), d) Depth (m), e) Mixed layer depth (MLD, in m), f) Age (days).

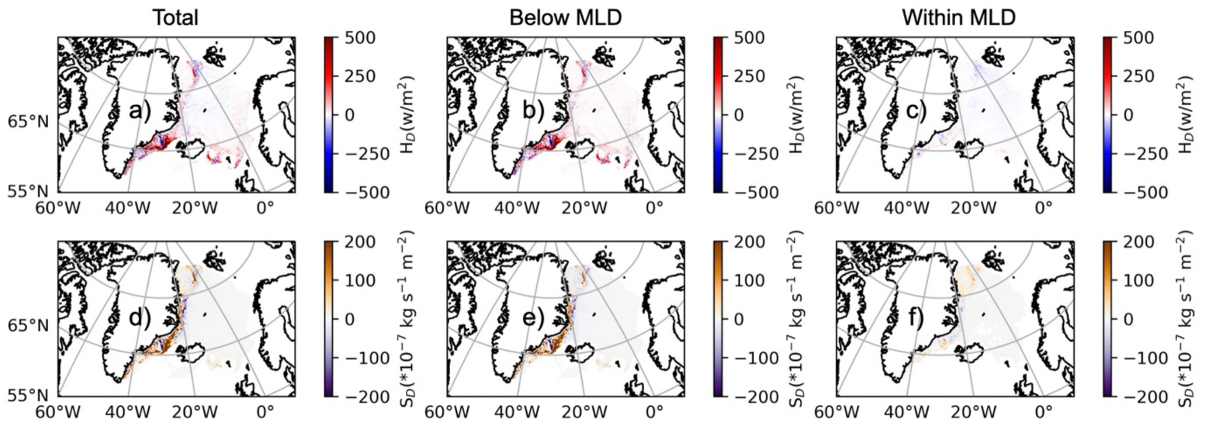

Supplementary Figure 2: Lagrangian heat divergence (a-c, calculated using equation 4) and salt divergence (d-f, calculated using equation 6) for the Arctic waters that started from the Fram Strait and reached at the eastern Subpolar North Atlantic Ocean (SPNA) section. 1st Column: Total divergence, 2nd column: divergence occurring below the mixed layer depth (MLD) and 3rd column: divergence within the MLD. Positive values indicate heat/salt gain and opposite holds true for negative values.

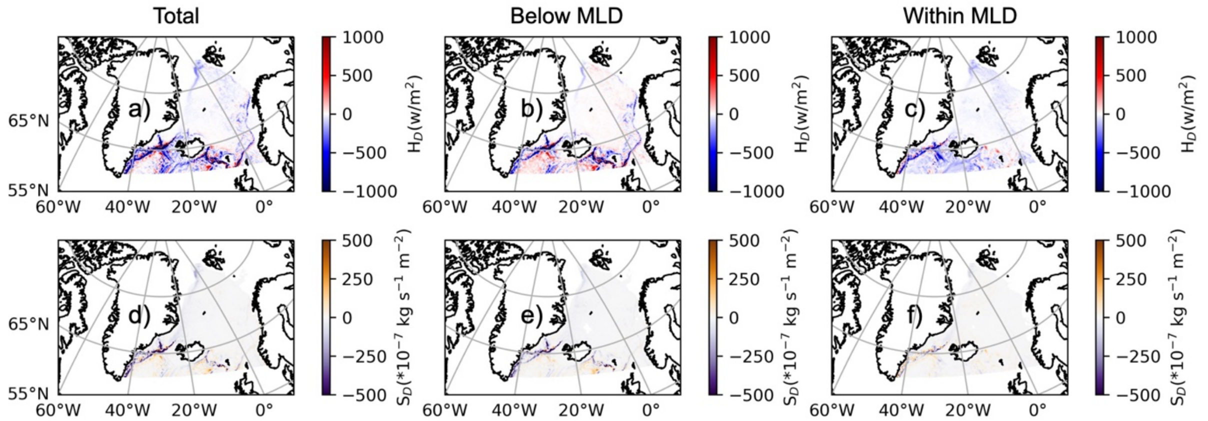

Supplementary Figure 3: Same as Supplementary Fig. 2 but for the Atlantic waters that started and ended at the eastern Subpolar North Atlantic Ocean section.

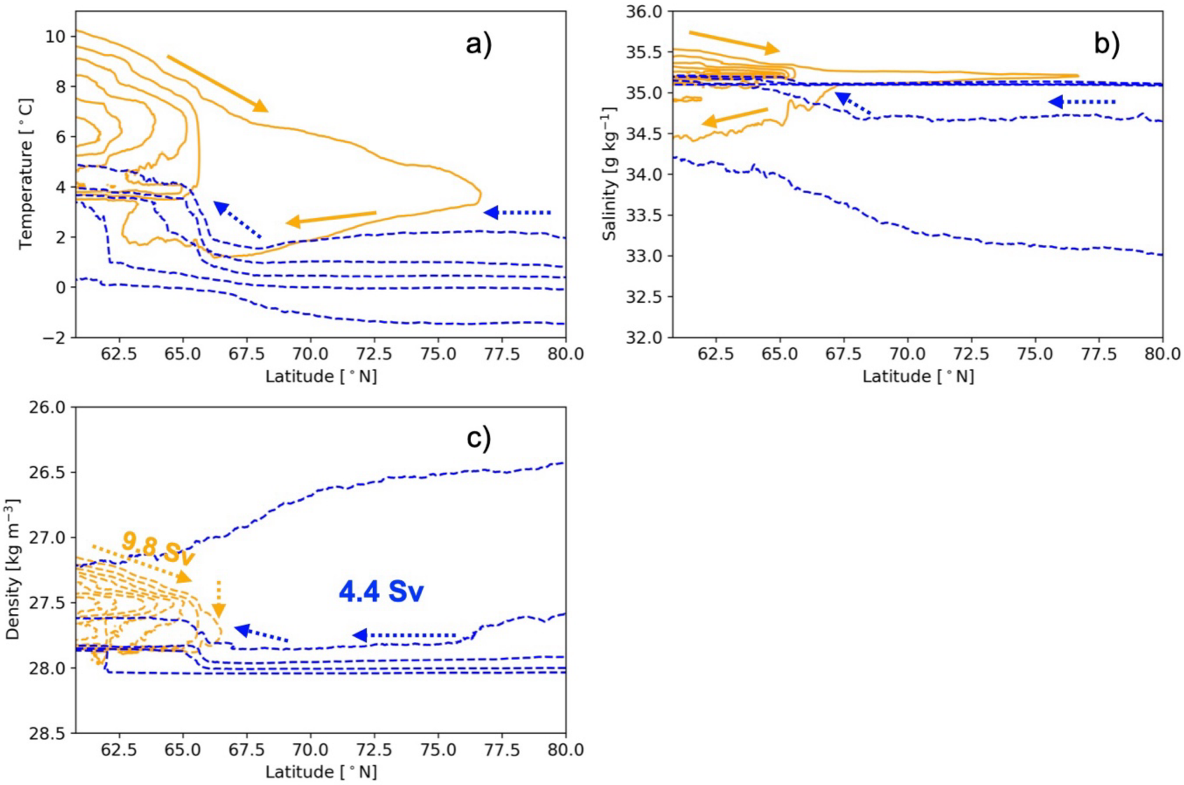

Supplementary Figure 4: Meridional overturning stream function in a) temperature-latitude, b) salinity-latitude and c) density-latitude coordinate system. This has been computed with backward trajectories that have started and exited at the eastern Subpolar North Atlantic Ocean (SPNA) section. The orange lines indicate Atlantic inflows and blue dashed lines correspond to Arctic waters that have reached the eastern SPNA. The direction of the water movement in each figure is indicated by arrows. The contour intervals are 2 Sv and starts at 1 Sv for solid contours. The dashed streamlines are plotted with 1 Sv interval and starts at -1 Sv. The Atlantic and Arctic water contributions to the Atlantic Meridional Overturning Circulation (AMOC) lower limb are shown with bold orange and blue numbers respectively. Note, the stream functions closely resemble with Figure 3c, which were obtained using the forward trajectory simulation.

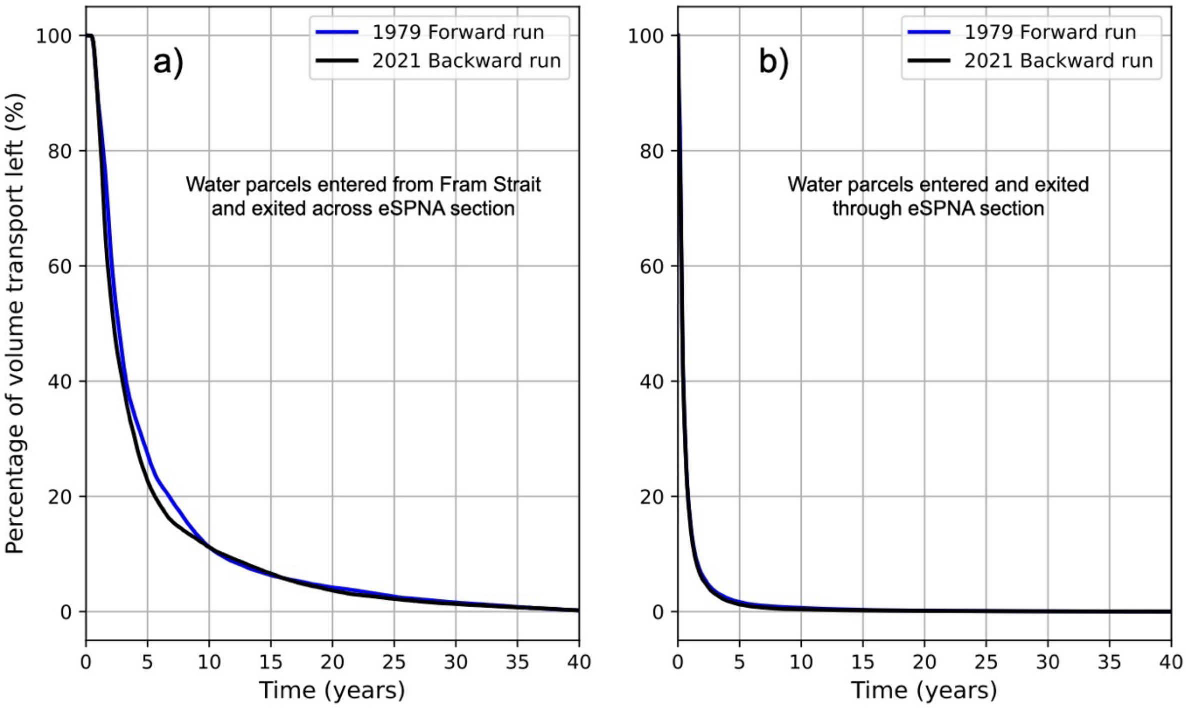

Supplementary Figure 5: Time taken by the percentage of water parcels that have a) entered from Fram strait and exited across eastern Subpolar North Atlantic Ocean (SPNA) section and b) entered and crossed through eastern SPNA section only.

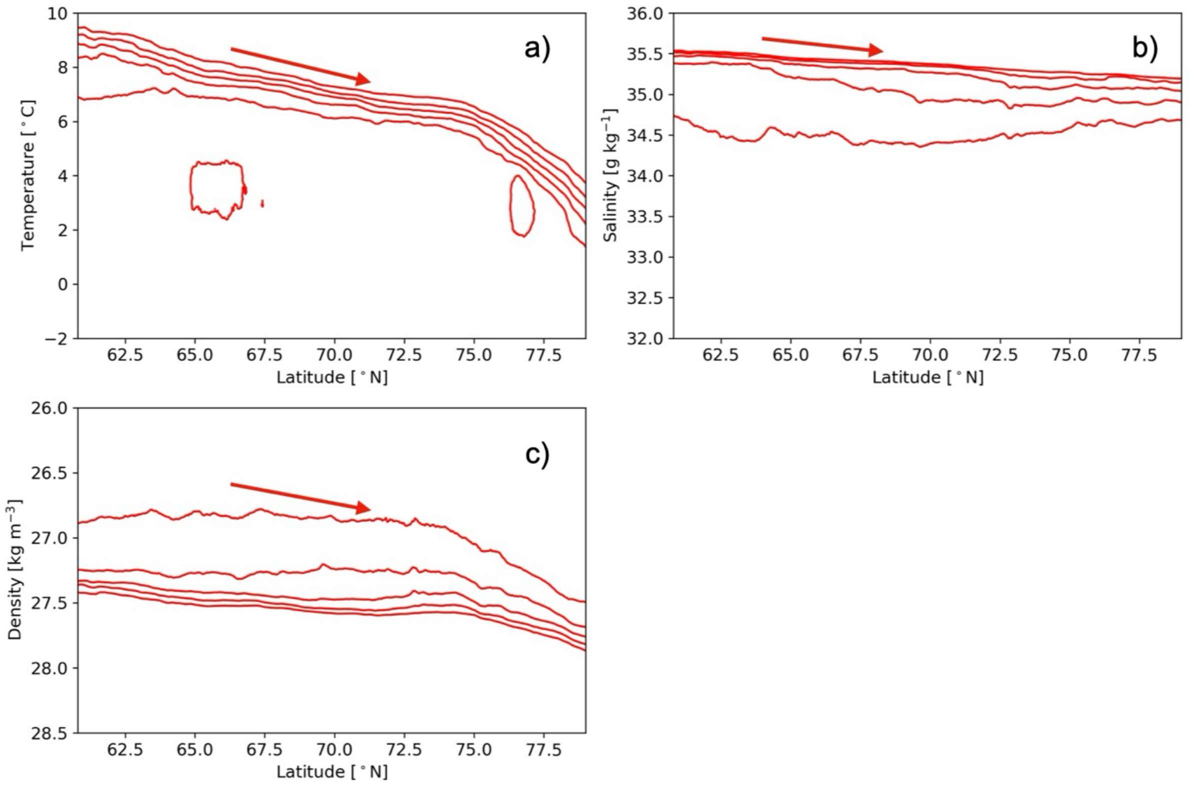

Supplementary Figure 6: Meridional overturning stream function in a) temperature-latitude, b) salinity-latitude and c) density-latitude coordinate system. This has been computed with forward trajectories that have started from the eastern Subpolar North Atlantic Ocean (SPNA) section and entered the Barents Sea. The direction of the water movement in each figure is indicated by arrows. The contour intervals are 1 Sv and starts at 0.5 Sv.

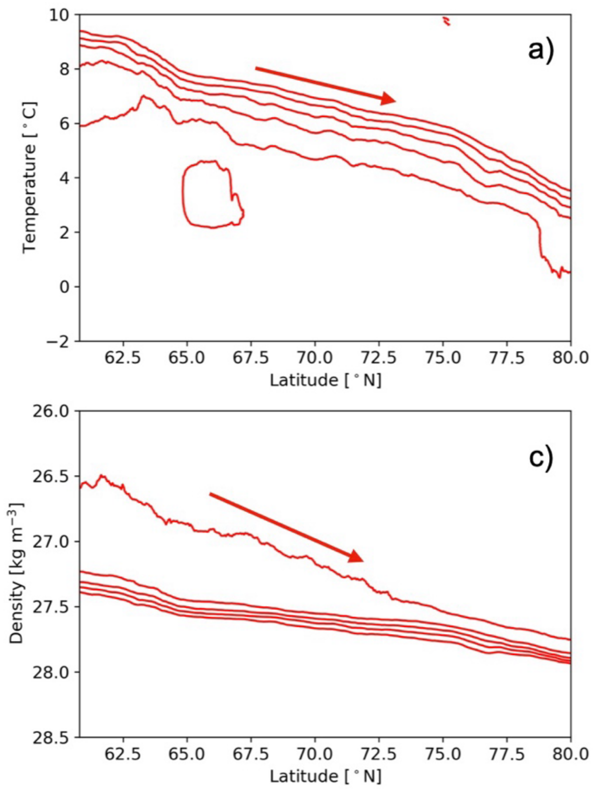

Supplementary Figure 7: Same as previous but computed with forward trajectories that have started from the eastern Subpolar North Atlantic Ocean (SPNA) section and crossed the Fram Strait. The direction of the water movement in each figure is indicated by arrows. The contour intervals are 1 Sv and starts at 0.5 Sv.
